# Detection of *Streptococcus pyogenes* in an atypical hematological diagnostic case

**DOI:** 10.1007/s15010-024-02335-5

**Published:** 2024-07-25

**Authors:** Enrico Schalk, Svea Genseke, Andreas E. Zautner, Achim J. Kaasch

**Affiliations:** 1 Department of Hematology, Oncology and Cell Therapy, Medical Faculty, Otto von Guericke University Magdeburg, Leipziger Str. 44, D-39120 Magdeburg, Germany; 2 Institute of Medical Microbiology and Hospital Hygiene, Medical Faculty, Otto von Guericke University Magdeburg, Magdeburg, Germany; 3 Health Campus Immunology, Infectiology and Inflammation (GCI), Medical Faculty, Otto von Guericke University Magdeburg, Magdeburg, Germany

**Keywords:** *Streptococcus pyogenes*, Pleural effusion, May-Grünwald-Giemsa staining, Gram staining, Morphology, Cytocentrifuged preparation, Blood culture

A sample of a native pleural effusion, taken from a 45-years old man with HIV and known Hodgkin lymphoma in complete remission, was submitted to the hematology laboratory for cytological examination regarding relapsed lymphoma. In a cytocentrifuged and May-Grünwald-Giemsa (MGG) stained preparation, lymphoma cells were not detected. Besides many mostly lytic granulocytes, many small, dark blue to black structures were observed. These were round to slightly elongated or ovoid shaped and located extracellularly or within granulocytes. Some of these structures were in pairs or in short chains (Fig. 1A). Thus, streptococci were suspected.

**Fig. 1:**
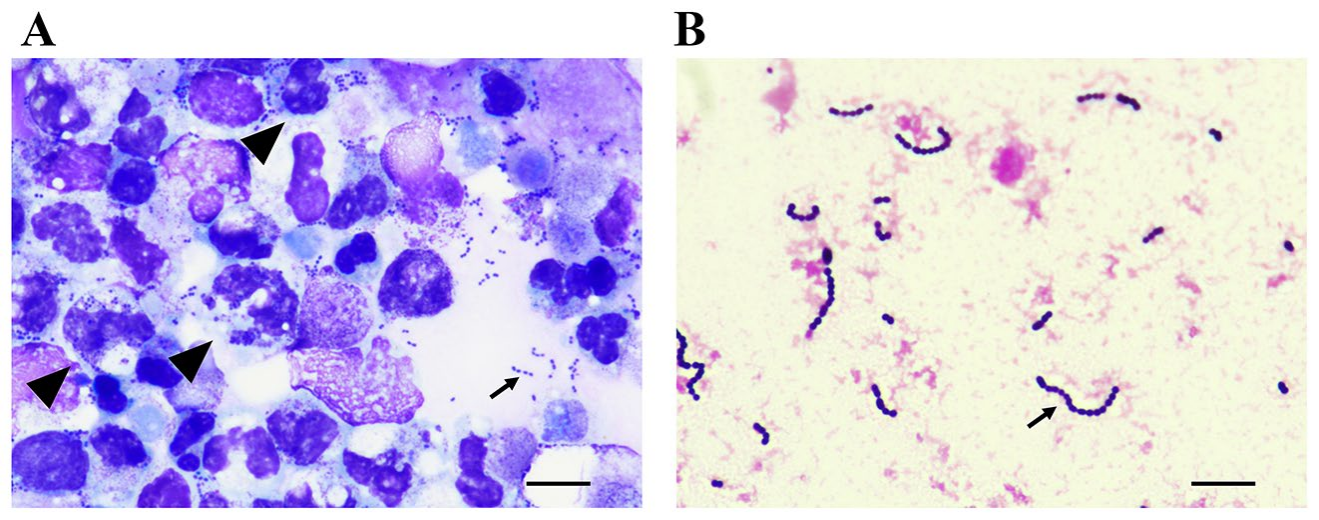
*Streptococcus pyogenes* in a pleural effusion. **A** Native, hematological-cytological cytocentrifuged preparation, May-Grünwald-Giemsa staining. Cell detritus, lytic leucocytes/granulocytes with intracellular cocci (arrow heads); and small extracellular cocci in pairs and short chains (arrow). In line with the molecular phenotype, a capsule appears to be visible around the bacteria. **B** Cultured in blood culture, Gram staining. Typical Gram-positive chain cocci (arrow) **Note.** Magnification 1000x in both panels; the bar indicates 10 μm in both panels

In the microbiology laboratory, an aerobic blood culture bottle inoculated with the pleural effusion yielded typical Gram-positive cocci (Fig. 1B), that were identified by the Bruker MALDI-Biotyper^®^ system as *Streptococcus pyogenes* (score value 2.34). Whole-genome sequencing (Illumina^®^ MiSeq™) revealed an intact *hasABC* operon responsible for the synthesis of a hyaluronic acid capsule (Fig. [1]A). M-protein gene typing resulted in emm92.0, ruling out the hypertoxigenic virulent M1_UK_ genotype [1].

MGG is the standard panoptic stain used for hematological-cytological preparations in Europe [2], but can also be utilized in microbiological diagnostics [3, 4]. *Streptococcus* spp. can be identified in MGG stained hematological-cytological preparations [5].

In contrast to this particular case, the observation of bacteria in a hematological diagnostic sample is quite rare. The variations in the morphological appearance of *S. pyogenes*, such as size, shape, and arrangement, may be influenced by factors like staining technique (MGG vs. Gram), growth media (native vs. cultured in blood culture vs. solid agar), and sample preparation (cytocentrifuged vs. non-cytocentrifuged).

## Data Availability

The whole-genome sequence of isolate *S. pyogenes* 318231 is available on NCBI GenBank^®^ with the following details: Accessions: CP154891 (chromosome), CP154892 (plasmid), BioProject: PRJNA1101912, BioSample: SAMN41005651.

## References

[1] Wolters M, Berinson B, Degel-Brossmann N, Hoffmann A, Bluszis R, Aepfelbacher M, Rohde H, Christner M. Population of invasive group a streptococci isolates from a German tertiary care center is dominated by the hypertoxigenic virulent M1_UK_ genotype. Infection. 2023;52:667–71. 10.1007/s15010-023-02137-1. 10.1007/s15010-023-02137-1 PMC10954911 38064158

[2] Binder T, Diem H, Fuchs R, Gutensohn K, Nebe T. Pappenheim stain: description of a hematological standard stain – history, chemistry, procedure, artifacts and problem solutions. J Lab Med. 2012;36:293–309. 10.1515/labmed-2012-0. [article in German].

[3] De Brauwer E, Jacobs J, Nieman F, Bruggeman C, Drent M. Test characteristics of acridine orange, Gram, and May-Grünwald-Giemsa stains for enumeration of intracellular organisms in bronchoalveolar lavage fluid. J Clin Microbiol. 1999;37:427–9. 10.1128/JCM.37.2.427-429.1999. 9889233 10.1128/jcm.37.2.427-429.1999 PMC84328

[4] Bousbia S, Raoult D, La Scola B. Pneumonia pathogen detection and microbial interactions in polymicrobial episodes. Future Microbiol. 2013;8:633–60. 10.2217/fmb.13.26. 23642118 10.2217/fmb.13.26

[5] Aue G, Austein T. *Streptococcus pneumoniae* sepsis in concurrently diagnosed multiple myeloma. Am J Hematol. 2007;82:858. 10.1002/ajh.20911. 17506071 10.1002/ajh.20911

